# Fractionation of an Extract of *Pluchea odorata* Separates a Property Indicative for the Induction of Cell Plasticity from One That Inhibits a Neoplastic Phenotype

**DOI:** 10.1155/2012/701927

**Published:** 2012-03-13

**Authors:** Mareike Seelinger, Ruxandra Popescu, Prapairat Seephonkai, Judith Singhuber, Benedikt Giessrigl, Christine Unger, Sabine Bauer, Karl-Heinz Wagner, Monika Fritzer-Szekeres, Thomas Szekeres, Rene Diaz, Foster M. Tut, Richard Frisch, Björn Feistel, Brigitte Kopp, Georg Krupitza

**Affiliations:** ^1^Institute of Clinical Pathology, Medical University of Vienna, Waehringer Guertel 18-20, 1090 Vienna, Austria; ^2^Department of Pharmacognosy, Faculty of Life Sciences, University of Vienna, AlthanstraBe 14, 1090 Vienna, Austria; ^3^Department of Nutritional Sciences, Faculty of Life Sciences, University of Vienna, AlthanstraBe 14, 1090 Vienna, Austria; ^4^Clinical Institute of Medical and Chemical Laboratory Diagnostics, Medical University of Vienna, Waehringer Guertel 18-20, 1090 Vienna, Austria; ^5^Institute for Ethnobiology, Playa Diana, San José, Petén, Guatemala; ^6^Finzelberg GmbH & Co. KG, Koblenzer Strasse 48-54, 56626 Andernach, Germany

## Abstract

*Introduction*. Several studies demonstrated that anti-inflammatory remedies exhibit excellent anti-neoplastic properties. An extract of *Pluchea odorata* (Asteraceae), which is used for wound healing and against inflammatory conditions, was fractionated and properties correlating to anti-neoplastic and wound healing effects were separated. *Methods*. Up to six fractionation steps using silica gel, Sephadex columns, and distinct solvent systems were used, and eluted fractions were analysed by thin layer chromatography, apoptosis, and proliferation assays. The expression of oncogenes and proteins regulating cell migration was investigated by immunoblotting after treating HL60 cells with the most active fractions. *Results*. Sequential fractionations enriched anti-neoplastic activities which suppressed oncogene expression of JunB, c-Jun, c-Myc, and Stat3. Furthermore, a fraction (F4.6.3) inducing or keeping up expression of the mobility markers MYPT, ROCK1, and paxillin could be separated from another fraction (F4.3.7), which inhibited these markers. *Conclusions*. Wound healing builds up scar or specific tissue, and hence, compounds enhancing cell migration support this process. In contrast, successful anti-neoplastic therapy combats tumour progression, and thus, suppression of cell migration is mandatory.

## 1. Introduction

Drug discovery is a constant need for biomedical research and clinical progress. Particularly mega-biodiversity areas such as the tropical rainforests of Central America are very promising for the discovery of new pharmaceutical lead compounds. Therefore, we chose an ethnomedical approach to drug discovery and focussed on traditional remedies of the Maya to preselect plants with proven health effects.

Traditional medicine plants are often used for hundreds of years [1], which is the reason why no or only little toxic effects in humans can be expected. Especially in Africa and Central and South America, traditional medicine is advised by shamans, curanderos, or herbalists who often keep the use of the healing plants as a secret [2]. Although these plants are used since ages, little is known about the “pinciples of activity” and/or the targeted cellular mechanisms, and hence, these remedies have a great potential for drug development. We aimed for the separation and isolation of distinct properties of the Maya healing plant *Pluchea odorata* which are relevant for anticancer treatment. *P. odorata* grows in the USA, Mexico, Belize, Guatemala, Panama, Cayman Islands, Guadeloupe, Jamaica, Puerto Rico, St. Lucia, Venezuela, and Ecuador (Germplasm Resources Information Network, United States Department of Agriculture, 9 November 2004, http://www.ars-grin.gov/cgi-bin/npgs/html/taxon.pl?104497) and is still used by the Maya to treat common cold, fever, flu, head colds, headache, hypertension, neuralgia, ophthalmia, palsy, pneumonia, snake bite, swellings, inflammation, and bruises of the skin [3]. The medical solution is prepared by boiling two handfuls of leaves in one gallon of water, and then it is frequently applied on the affected area until the inflammation subsides [4]. Further the plant is described as being antidote, astringent, diaphoretic, and haemostatic [5], as well as traditionally used by mothers after giving birth to decrease the risk for infections and conveyance of tissue recovery [3]. Gridling et al. [6] and Bauer et al. [7] describe a strong anti-neoplastic effect of the dichloromethane extract in HL60 and MCF-7 cells and an anti-inflammatory response in HUVECs (human umbilical vein endothelial cells). 

Pharmaceutical drugs are prepared under standardised conditions and usually contain just one active principle. In contrast, ethnopharmacologic remedies are mixtures of a vast number of compounds, which are in many cases unknown, and vary in their composition and activity depending on the growth area and the time of collection. This makes them difficult for application and trading. Moreover, plant extracts can also contain compounds that are systemically toxic or counteract the desired effect of the active principle(s). On the other hand, some of the different compounds in a plant extract can synergise. The present work will give examples of this phenomenon. A common reason to terminate *a priori* successful therapies of all kinds of cancer and other diseases is the acquisition of drug resistance. However, it has not been reported so far that complex plant mixtures induce treatment resistances in patients, most likely because several distinct active principles target various intra- and intercellular signalling pathways simultaneously thereby preventing that the organism develops an “escape mechanism.” Thus, the development of complex extracts should be considered as a strategy to treat cancer. Standardisation procedures for such mixtures must be individually developed to provide therapeutics which are effective and safe and do not exhibit batch to batch variations. The fractionation and accompanying testing in bioassays and by appropriate analyses are a feasible concept for standardisation and remedies emerging along such procedures can be approved by national agencies (i.e., Avemar, WO 2004014406 (A1): “Use of a fermented wheat germ extract as anti-inflammatory agent” by Hidvegi and Resetar).

Here we describe a fractionation approach of the dichloromethane extract of *P. odorata*, which was constantly controlled regarding its activity by bio-assays and analyses that can be standardised. This resulted in fractions causing distinct cellular responses indicative for wound healing or anti-neoplastic properties.

## 2. Material and Methods

### 2.1. Plant Material

The aerial parts (leaves, caulis, florescence) of *Pluchea odorata* (L.) Cass. were collected in Guatemala, Departamento Petén, near the north-western shore of Lago Petén Itzá, San José, within an area of four-year-old secondary vegetation ~1 km north of the road from San José to La Nueva San José (16°59′30′′ N, 89°54′00′′ W). Voucher specimens (leg. G. Krupitza & R. O. Frisch, Nr. 1-2009, 08. 04. 2009, Herbarium W) were archived at the Museum of Natural History, Vienna, Austria. 6 kg of air-dried plant material has been extracted with dichloromethane by Björn Feistel (Finzelberg GmbH & Co. KG, Andernach, Germany), and the extract stored in an exsiccator in the dark at 4°C until use.

### 2.2. Plant Extraction and Fractionation

Plant extraction with dichloromethane was essentially as described earlier [6, 7]. 6000 g dried plant material was extracted with dichloromethane resulting in 220 g dry extract (extraction was performed by Finzelberg GmbH & Co. KG, Andernach, Germany).

 Vacuum Liquid Chromatography (VLC) was used for the separation of large amounts of extract. 36 g dichloromethane extract was redissolved in dichloromethane, mixed with 70 g silica gel and evaporated to dryness, and ground in a mortar to obtain a homogenous powder. A 12 × 40 cm column was packed with 900 g silica gel, on top the silica gel-containing extract, and covered with sea sand to ballast the sample. The mobile phase was passed through by application of reduced pressure. After checking the collected fractions by TLC, those with similar bands were recombined, and this resulted in three combined fractions (F1.1–F1.3; Table 1).

*VLC Fractionation of F1.1*. 10 g of F1.1, which was derived from the crude extract, was dissolved in dichloromethane, mixed with 20 g silica gel, evaporated to dryness, refined in a mortar, and applied on top of a 5 × 60 cm silica gel column. Compounds were eluted with the mobile phases shown in Table 2 by applying reduced pressure. Collected fractions were checked by TLC, and similar fractions were recombined yielding nine combined fractions (F2.1–F2.9).

 Column Chromatography (CC) Fractionation of F2.6 (Less Than 2 g of Residue was fractionated by CC). 1.6 g F2.6, which was gained by fractionation of F1.1 was dissolved in dichloromethane, mixed with 3 g silica gel, evaporated to dryness, placed on top of a 5 × 50 cm silicia gel column and eluted with mobile phases shown in Table 3. The collected fractions were checked by TLC and those with similar band patterns were recombined to yield ten combined fractions (F3.1–F3.10).

 CC Fractionation of F3.3. 1.08 g of F3.3, which was derived from F2.6, was dissolved in dichloromethane and placed on top of a 2 × 30 cm silica gel column. Mobile phases were used as illustrated in Table 4. Fractions were checked by TLC, and those with similar band patterns were recombined yielding twelve combined fractions (F4.3.1–F4.3.12).

 CC Fractionation of F4.3.7. 146 mg F4.3.7 was dissolved dichloromethane and applied on a 80 × 1.5 cm silica gel column and fractionated with one litre solvent (chloroform: methanol: water, 95 : 1.5 : 0.1). Fractions were collected in tubes, ten drops per minute, and every 30 minutes the tubes were changed. Afterwards the column was washed with 300 mL methanol. Fractions were checked by TLC, and those with similar band patterns were recombined resulting in twelve fractions (F5.3.7.1–F5.3.7.12; see Figure 3(c)).

 CC Fractionation of F3.6. 0.32 g F3.6, which was derived from F2.6, was dissolved in methanol and applied on top of a 3.5 × 40 cm Sephadex column (2.5 × 40 cm for 3.6). Methanol was used as mobile phase, and fractions were collected and checked by TLC, and those with similar band patterns were recombined to yield four combined fractions: F4.6.1 (9 mg), F4.6.2 (5 mg), **F4.6.3** (207 mg), F4.6.4 (55 mg).

 CC Fractionation of F4.6.3. 20 mg F4.6.3 (very oily), which was derived from F3.6, was dissolved in dichloromethane, mixed with silica gel, and applied on top a 2.5 × 15 cm dichloromethane conditioned silica gel column. Successful elution was achieved with dichloromethane : ethylacetate (50 : 50). Fractions were checked by TLC and those with similar band patterns were recombined yielding six combined fractions (F5.6.3.1–F5.6.3.6; see Figure 4(b)).

### 2.3. Thin Layer Chromatography (TLC)

TLC was used for control of fractionation. Stationary phase and mobile phases are described in Table 5. The mobile phase varied between five solvent systems. Plates were detected under UV_254_, UV_366_, and visible light, before and after spraying with anisaldehyde sulphuric acid reagent (ASR). ASR consisted of 0.5 mL anisaldehyde, 10 mL glacial acetic acid, 85 mL methanol and 5 mL H_2_SO_4_ (sulfuric acid). The sprayed plate was heated at 100°C for five minutes, and then compounds were detected under UV_254,365_ and visible light. Unless otherwise stated 8 *μ*L extract or fraction solution was applied to the plate.

### 2.4. Cell Culture

HL-60 (human promyelocytic leukaemia cell) cells were purchased from American Type Culture Collection (ATCC). The cells were grown in RPMI 1640 medium which was supplemented with 10% heat-inactivated fetal calf serum (FCS), 1% Glutamax, and 1% Penicillin-Streptomycin. Both medium and supplements were obtained from Life Technologies (Carlsbad, CA, USA). The cells were kept in humidified atmosphere at 37°C containing 5% CO_2_.

### 2.5. Proliferation Assay

Proliferation assays [8, 9] were performed to analyse the inhibition of proliferation of HL-60 cells treated with extract or fractions of *P. odorata*. Extract and fractions were dissolved in ethanol (final concentration was 0.2%). HL-60 cells were seeded in 24-well plates at a concentration of 1 × 10^5^ cells per mL RPMI medium allowing logarithmic growth within the time of treatment with plant extract or fractions. The control was treated with solvent. After 24 and 48 hours, the number of cells was determined using the Sysmex Cell Counter (Sysmex Corp., Kobe, Japan). Experiments were done in triplicate. Percentage of cell division progression compared to the untreated control was calculated by applying the following formula:

(1) $$\frac{{\text{C}}_{\text{48 or 72 h}}+\;\text{drug}-{\text{C}}_{\text{24 h}}+\;\text{drug}}{{\text{C}}_{\text{48 or 72 h}}-\;\text{drug}-{\text{C}}_{\text{24 h}}+\;\text{drug}}\times 100=\%\;\text{cell division}.$$

### 2.6. Apoptosis Assay

Determination of cell death by Hoechst 33258 (HO) and propidium iodide (PI) double staining (both Sigma, St. Louis, MO, USA) allows identifying the amount and the type of cell death (early or late apoptosis or necrosis) [10–12]. Therefore, HL-60 cells were seeded in a 24-well plate at a concentration of 1 × 10^5^ cells per mL RPMI medium. Cells were treated with fractions or extract of *P. odorata.* The cells were incubated for 8, 24, 48, and/or 72 hours, depending on the experiment. At each time point, 100 *μ*L cell suspension of each well was transferred into separate wells of a 96-well plate, and Hoechst 33285 and propidium iodide were added at final concentrations of 5 *μ*g/mL and 2 *μ*g/mL, respectively. After one hour of incubation at 37°C, stained cells were examined and photographed with an Axiovert fluorescence microscope (Zeiss, Jena, Germany) equipped with a DAPI filter. Type and number of cell deaths were evaluated by visual examination of the photographs according to the morphological characteristics revealed by HOPI staining. Experiments were done in triplicate.

### 2.7. Western Blotting

Preparation of lysates. HL60 cells were seeded in a tissue culture flask at a concentration of 1 × 10^6^ cells per mL RPMI medium. *P. odorata* fractions F1, F4.6.3, and F4.3.7 were analysed by western blots. HL60 cells were either incubated with 40 *μ*g/mL F1 or with 10 *μ*g/mL of one of the other two *P. odorata* fractions for the indicated times. At each time point, 4 × 10^6^ cells were harvested, placed on ice, and centrifuged (1000 rpm, 4°C, 4 min). Then, the supernatant (medium) was discarded, and the pellet was washed twice with cold phosphate buffered saline (PBS, pH 7.2) and centrifuged (1000 rpm, 4°C, 4 min). The cell pellet was lysed in a buffer containing 150 mM NaCl, 50 mM Tris (pH 8.0), 1% Triton-X-100, 1 mM phenylmethylsulfonyl fluoride (PMSF), and 1 mM protease inhibitor cocktail (PIC) (Sigma, Schnelldorf, Germany). Afterwards the lysate was centrifuged at 12000 rpm for 20 min at 4°C. Supernatant was transferred into a 1.5 mL tube and stored at −20°C for further analyses.

Gel electrophoresis (SDS-PAGE) and blotting. Equal amounts of protein samples (lysate) were mixed with sodium dodecyl sulfate (SDS) sample buffer (1 : 1) and loaded onto a 10% polyacrylamide gel. Proteins were separated by polyacrylamide gel electrophoresis (PAGE) at 120 Volt for approximately one hour. To make proteins accessible to antibody detection, they were electrotransferred from the gel onto a polyvinylidene difluoride (PVDF) Hybond membrane (Amersham, Buckinghamshire, UK) at 95 Volt for 80 minutes. Membranes were allowed to dry for 30 minutes to provide fixing of the proteins on the membrane. Methanol was used to moist the membranes. Equal sample loading was checked by staining the membrane with Ponceau S (Sigma, Schnelldorf, Germany).

Protein detection. After washing with PBS or TBS (Tris buffered saline, pH 7.6), membranes were blocked in PBS- or TBS-milk (5% nonfat dry milk in PBS containing 0.5% Tween 20 or TBS containing 0.1% Tween 20) for one hour at room temperature. Then membranes were washed with PBS/T (PBS containing 0.5% Tween 20) or TBS/T (TBS containing 0.1% Tween 20), changing the washing solution four to five times every five minutes. Then every membrane was incubated with a primary antibody (1 : 500) in blocking solution (according to the data sheet TBS-, PBS-milk or TBS-, PBS-BSA), at 4°C over night gently shaking. Subsequently the membrane was again washed with PBS/T or TBS/T and incubated with the second antibody (peroxidase-conjugated goat anti-rabbit IgG or anti-mouse IgG) diluted 1 : 2000 for one hour at room temperature. After washing the membranes, chemiluminescence was developed with enhanced chemiluminescence (ECL) plus detection kit (Amersham, UK) (two seconds to ten minutes), and membranes were exposed to the Lumi-Imager F1 (Roche) for increasing times.

### 2.8. Antibodies

Monoclonal mouse ascites fluid anti-acetylated *α*-tubulin (6-11B-1) and *β* actin (AC-15) antibodies were from Sigma (St. Louis, MO, USA). Monoclonal mouse *α*-tubulin (DM1A), *β*-tubulin (H-235), Cdc25A (F-6), and polyclonal rabbit paxillin (H-114), ROCK-1 (C8F7), c-Jun (H-79), Jun b (210) were from Santa Cruz Biotechnology, Inc. (Santa Cruz, CA, USA). Monoclonal rabbit cleaved caspase 8 (Asp391) (18C8) and phospho-Stat3 (Tyr705)(D3A7) antibodies and polyclonal rabbit cleaved caspase 3 (Asp175), human-specific cleaved caspase 9 (Asp330), phospho-Chk2 (Thr68), Chk2, phospho-myosin light chain 2 (MLC2-Ser19), myosin light chain 2, Stat3, and MYPT1 were from Cell Signaling (Danvers, MA, USA). Polyclonal rabbit phospho-Cdc25A (Ser177) antibody was from Abgent (San Diego, CA, USA), polyclonal rabbit phospho-MYPT1 (Thr696) from Upstate (NY, USA), mouse monoclonal *γ*H2AX (pSer139) (DR 1017) from Calbiochem (San Diego, CA, USA), and mouse monoclonal c-Myc Ab-2 (9E10.3) from Thermo Fisher Scientific, Inc. (Fremont, CA, USA). The secondary antibodies peroxidase-conjugated anti-rabbit IgG and anti-mouse IgG were purchased from Dako (Glostrup, Denmark).

### 2.9. Statistical Analysis

For statistical analyses, Excel 2003 software and Prism 5 software package (GraphPad, San Diego, CA, USA) were used. The values were expressed as mean ± standard deviation, and Student's *t*-test was applied to compare differences between control samples and treatment groups. Statistical significance level was set to *P* < 0.05.

## 3. Results and Discussion

**Figure 1:**
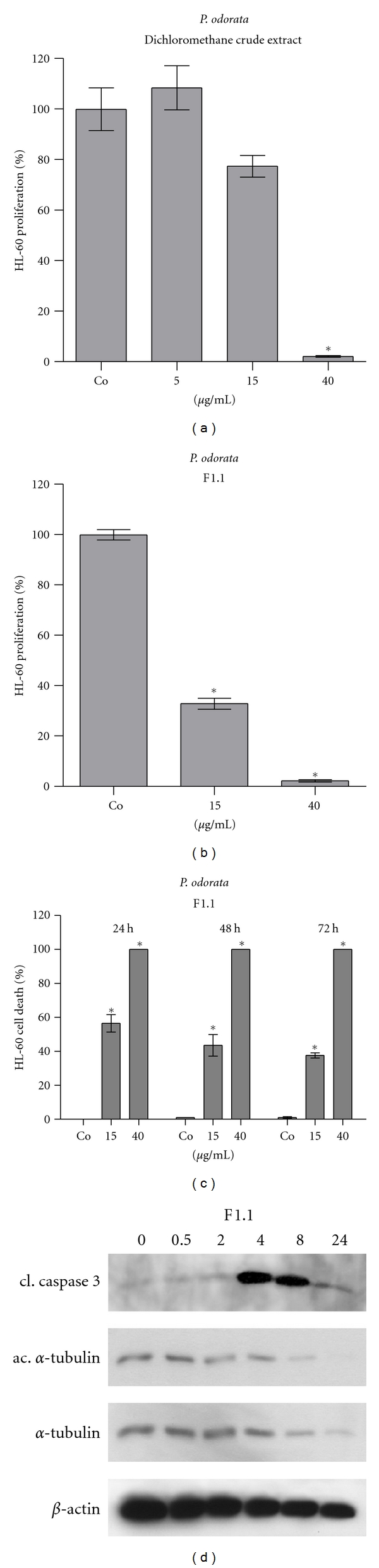
Antiproliferative effect of (a) dichloromethane crude extract, (b) fraction F1.1 in HL60 cells; 1 × 10^5^ cells/mL were seeded into 24-well plate, incubated with 5, 15, and 40 *μ*g/mL extract or fraction F1.1, and the percentage of proliferation was calculated relative to solvent treated control within a 24 h period, (c) Induction of apoptosis by F1.1: cells were grown as described and incubated with 15 and 40 *μ*g/mL of each fraction for 72 h. Then, cells were double stained with Hoechst 33258 and propidium iodide and examined under the microscope with UV light connected to a DAPI filter. Nuclei with morphological changes which indicated cell death were counted, and the percentages of dead cells were calculated. Experiments were performed in triplicate. Asterisks indicate significance compared to untreated control (*P* < 0.05), and error bars indicate ±SD. (d) 1 × 10^6^ cells/mL were incubated with 40 *μ*g/mL F1.1 and harvested after 0.5, 2, 4, 8, and 24 h of treatment, lysed, and total protein applied to SDS-PAGE. Western blot analysis was conducted with the indicated antibodies. Equal sample loading was confirmed by Ponceau S staining and *β*-actin analysis.

The dichloromethane extract of *P. odorata* exhibits strong anti-neoplastic activity (Figure 1(a)). Therefore, we fractionated this extract and constantly monitored the activities by bio-assays measuring apoptosis and/or proliferation rates to get closer to the active principles. In the first round the crude extract was split in three distinct fractions of which fraction F1.1 exhibited the strongest anti-proliferative and proapoptotic activity (Figures 1(b) and 1(c)). 40 *μ*g F1.1/mL induced caspase 3 and decreased *β*-actin and *α*-tubulin levels, and therefore also reduced acetylation levels of *α*-tubulin were detected within 4 h of treatment (Figure 1(d)). This suggested that the overall protein decrease was due to the early activation of caspase 3 and subsequent cell death. 

**Figure 2:**
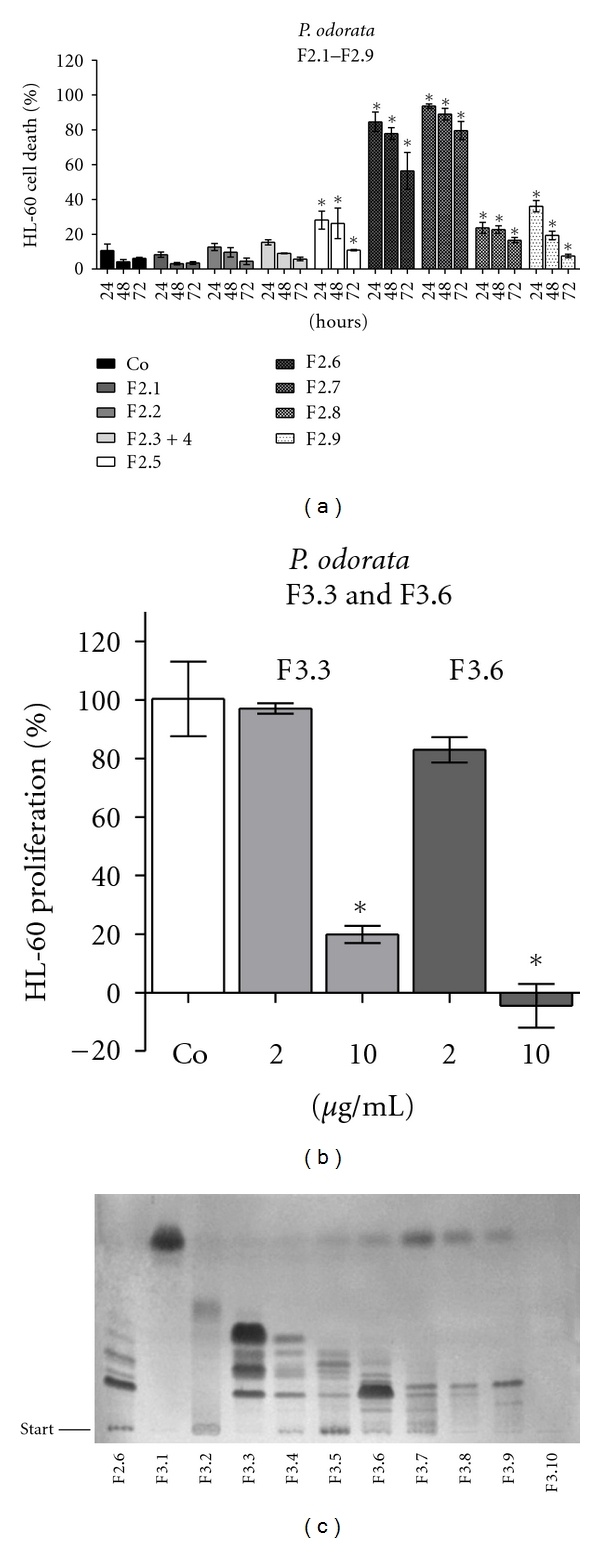
(a) Induction of apoptosis of HL60 cells by F2.1–F2.9. 1 × 10^5^ cells/mL were seeded in 24-well plates and incubated with 10 *μ*g/mL of each fraction for 72 h. Then, cells were double stained with Hoechst 33258 and propidium iodide and examined under the microscope with UV light connected to a DAPI filter. Nuclei with morphological changes which indicated cell death were counted, and the percentages of dead cells were calculated. Significance was calculated in comparison to control (Co). (b) Antiproliferative effect of F3.3 and F3.6: 1 × 10^5^ cells/mL were seeded into 24-well plate, incubated with 2 and 10 *μ*g/mL of each fraction, and the percentage of proliferation was calculated relative to solvent treated control within a 24 h period. Experiments were performed in triplicate. Asterisks indicate significance compared to untreated control (*P* < 0.05) and error bars indicate ±SD. (c) Thin layer chromatography (TLC) of F2.6 and F3.1–F3.10; Mobile phase: TLC system 2. Detection: visible light with ASR.

F1.1 was further processed yielding fractions F2.1–F2.9. F2.6 was nearly as active F2.7 (Figure 2(a)) but contained ~10 times more fraction material (1.9 g versus 0.2 g, resp.). Therefore, F2.6 was further processed yielding fractions F3.1–F3.10 (Figures 2(b) and 2(c)). 

10 *μ*g/mL of F3.4–F3.6 exhibited potent anti-proliferative properties in HL60 cells suppressing cell growth by 100%. The TLC analysis showed that both, F3.3 and F3.6, contained a distinct main compound, and therefore F3.3 and F3.6 were further fractionated. Also F3.2 was processed, however, this yielded only several low-activity fractions (data not shown).

**Figure 3:**
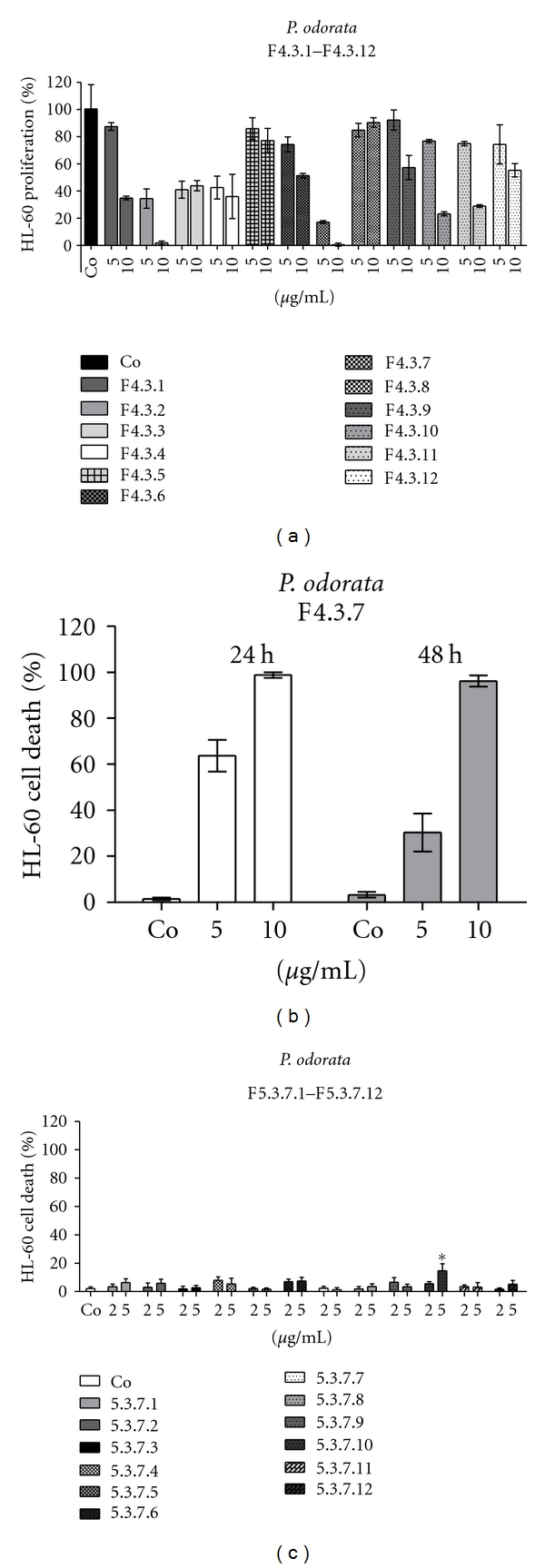
(a) Antiproliferative effect of F4.3.1–F4.3.12 in HL60 cells; 1 × 10^5^ cells/mL were seeded into 24-well plates, incubated with 5 and 10 *μ*g/mL of each fraction, and the percentage of proliferation was calculated relative to solvent treated control within a 24 h period. (b) Induction of apoptosis by F4.3.7 and (c) by F5.3.7.1–F5.3.7.12 in HL60 cells; 1 × 10^5^ cells/mL were seeded in 24-well plates and incubated with (b) 5 and 10 *μ*g/mL of F4.3.7 for 24 and 48 h and (c) with 2 and 5 *μ*g/mL of each indicated fraction for 24 h. Then, cells were double stained with Hoechst 33258 and propidium iodide and examined under the microscope with UV light connected to a DAPI filter. Nuclei with morphological changes which indicated cell death were counted, and the percentages of dead cells were calculated. Experiments were performed in triplicate. Asterisks indicate significance compared to untreated control (*P* < 0.05), and error bars indicate ±SD.

**Figure 4:**
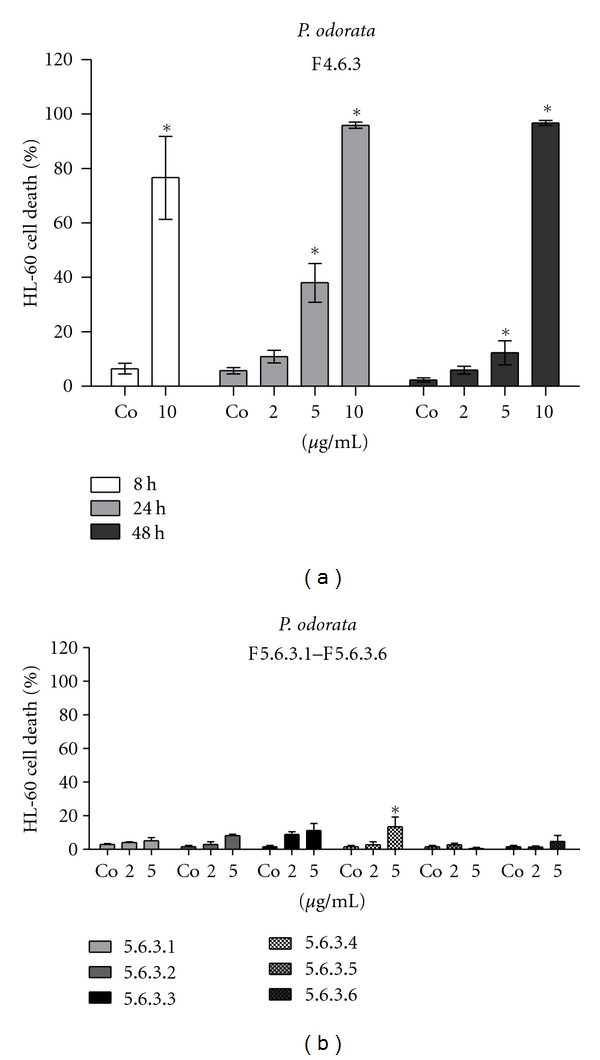
(a) Induction of apoptosis; 1 × 10^5^ HL60 cells/mL were seeded in 24-well plates and incubated with 2 and/or 10 *μ*g/mL of F4.6.3 for 8, 24, and 48 h or (b) 2 and 5 *μ*g/mL of F5.6.3.1–F5.6.3.6 for 24 h. Then, cells were double stained with Hoechst 33258 and propidium iodide and examined under the microscope with UV light connected to a DAPI filter. Nuclei with morphological changes which indicated cell death were counted, and the percentages of dead cells were calculated. Experiments were performed in triplicate. Asterisks indicate significance compared to untreated control (*P* < 0.05), and error bars indicate ±SD.

- *The subfractionation of F3.3* on a silica gel column yielded the potent antiproliferative and proapoptotic fraction F4.3.7 (Figures 3(a) and 3(b)). Further fractionation of F4.3.7 caused the decomposition of the strong cytotoxic activity into many low active fractions (F5.3.7.1–F5.3.7.12; Figure 3(c)), which in sum approximated the activity of the precursor.
- *The subprocessing of F3.6* yielded fractions F4.6.1–F4.6.4. F4.6.3, which was very oily, was the most pro-apoptotic one (Figure 4(a)). Upon further fractionation, the activity of F4.6.3 also decomposed into several low-activity fractions (F5.6.3.1–F5.6.3.6; Figure 4(b)).

**Figure 5:**
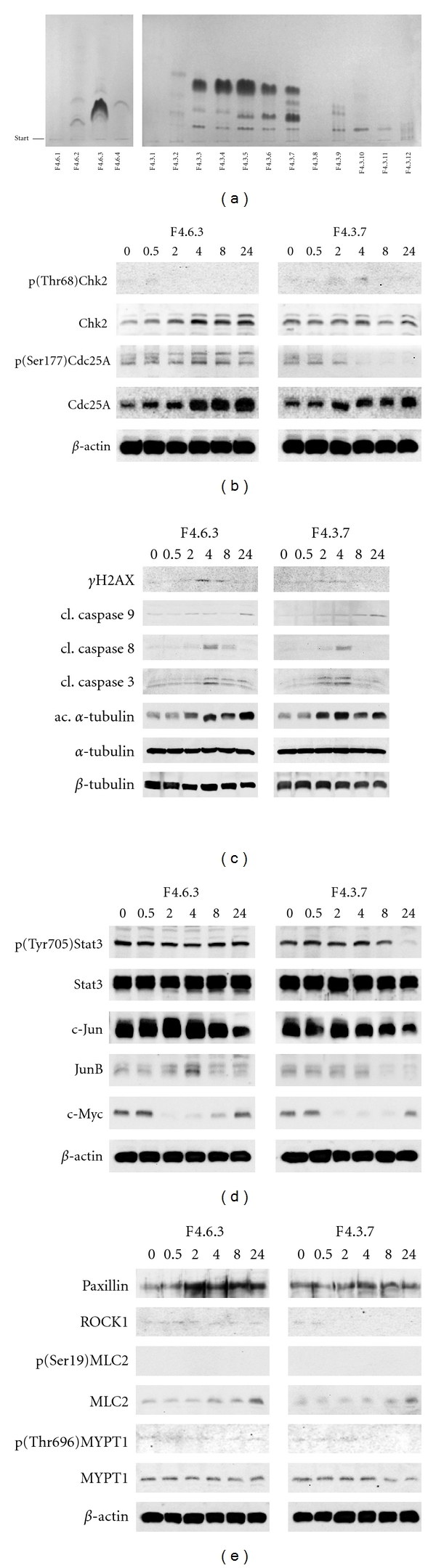
(a) Thin layer chromatography (TLC) of F4.6.3 (left panel) and F4.3.7 (right panel); mobile phase: TLC system 2; detection: UV_254_. Analysis of (b) cell cycle and checkpoint regulators, (c) apoptosis-related proteins, (d) oncogenes, (e) proteins required for mobility; 1 × 10^6^ HL60 cells/mL were incubated with 10 *μ*g/mL F4.6.3 and F4.3.7, respectively, and harvested after 0.5, 2, 4, 8, and 24 h of treatment. Cells were lysed and obtained proteins samples applied to SDS-PAGE. Western blot analysis was performed with the indicated antibodies. Equal sample loading was confirmed by Ponceau S staining *β*-actin or *β*-tubulin analysis.

Therefore, we went back to F4.3.7 and F4.6.3 and continued analyses with these two distinct high-activity fractions. The TLC patterns of F4.3.7 and F4.6.3 were clearly different from each other evidencing that they contain different compounds (Figure 5(a)). To characterise the two distinct fraction types, posttranslational modifications and expression levels of proteins, which are relevant for apoptosis and cell cycle arrest, were investigated. F4.3.7 slightly induced Chk2 phosphorylation and hence its activation, whereas F4.6.3 did not (Figure 5(b)). Chk2 was shown to phosphorylate Cdc25A at Ser177 and tags it for proteasomal degradation [13, 14]. However, treatment of HL60 cells with F4.3.7 caused the dephosphorylation of Ser177 and protein stabilisation. Also F4.6.3 stabilised Cdc25A despite Ser177 phosphorylation. This implicated that Cdc25A was not regulated by Chk2 activity in this scenario. Moreover Cdc25A stabilisation suggested an increase in cell cycle activity [15]. Nevertheless, cell proliferation was inhibited. 

In search of molecular causes for reduced proliferation, we found that the fractionation steps enriched a spindle toxin or an indirect microfilament-targeting activity. This was reflected by induced *α*-tubulin acetylation, which is an indicator of the polymerisation status of tubulin microfilaments [16, 17], within 2 h of treatment with F4.3.7 and F4.6.3 (Figure 5(c)). Thereafter, caspase 3 became activated. Also the phosphorylation of H2AX occurred after treatment with both fractions indicating DNA damage presumably due to caspase-3-induced DNase activity of DNA fragmentation factor (DFF; [18]), because caspase 3 activation and H2AX phosphorylation appeared simultaneously [19]. Evidently, the molecular onset of apoptosis occurred faster than the orchestrated expression of the investigated cell cycle regulators. Interestingly, the activation of caspase 3 concurred with the activation of caspase 8, but not with activation of caspase 9, indicating that the extrinsic apoptosis pathway became activated and not the intrinsic one. The signature-type processing of caspase 3 into the active fragment was more prominent by F1.1 than by F4.3.7 and F4.6.3. This was most likely due to the four times higher F1.1 concentration used (40 *μ*g/mL), which caused also the degradation of *α*-tubulin and *β*-actin as consequence of swift onset of cell death.

The low or absent activation of Chk2 (resp.) suggested that a genotoxic activity, which was formerly present in the crude extract [6] and in other subfractions [7], was eliminated throughout the described fractionations. Avoiding genotoxicity can be beneficial, because it reduces DNA damage and subsequent mutations that may cause secondary malignancies. Cdc25A, a classified oncogene [20, 21] was upregulated by both fraction types and therefore we investigated also the expression of other oncogenes, which are related to tumour growth and progression. F4.6.3 had a substantial effect on the repression of c-Myc and only a minor effect on c-Jun. Even more effective F4.3.7 suppressed also the phosphorylation of Tyr705 of Stat3 and thus inhibited its function, which is known to play a role in tumour progression [22]. This fraction further repressed JunB, c-Jun, and c-Myc more effectively than F4.6.3.

 Since *P. odorata* is used also as a wound healing remedy, this property also involves tissue regeneration and the regulation of a process called “epithelial to mesenchymal transition” (EMT) [23, 24]. The most prominent feature of EMT is the acquisition of a mobile phenotype [25, 26]. If transient and tightly regulated, EMT is beneficial for the organism, because it contributes to acute inflammation and tissue repair [27–29]. In contrast, the chronic status occurrence of EMT causes pathologies such as progression of cancer [30]. The mobility of cancer cells is a prerequisite for the intra- and extravasation of the vasculature, tissue invasion, and metastatic spread [23, 31], and it is mediated by proteins that allow cell plasticity and movement. Therefore, the alteration of motility-related gene products in cancerous cells, such as paxillin [32, 33], ROCK1 [34], MLC2 for the formation of stress fibres [35] and MYPT [36], are indicators for increased mobility and hence cancer progression. Also leukaemia cells such as differentiated HL60 attach to the ECM and vascular cells and transmigrate through vessel walls, invade tissues [37–39], and add to the progression of the disease. Hence, HL60 cells possess a repertoire of proteins facilitating cell movement although they normally grow in suspension. Treatment with F4.3.7 caused ROCK1 repression below constitutive and detectable levels, which was not the case with F4.6.3 (Figure 5(d)). Also MYPT expression decreased upon treatment with F4.3.7. Paxillin became upregulated by F4.6.3 treatment and marginally by F4.3.7, and this was also the case for the phosphorylation of MLC2. Since *P. odorata* is successfully used for tissue recovery and healing of skin bruises, the increased mobility of cells (fibroblasts, epithelial cells, macrophages, etc.) is required to close the tissue disruption. This very property, however, is detrimental throughout cancer treatment, and elimination of this activity might be beneficial for this purpose. Therefore, future research has to address the question whether F4.3.7 is advantageous for cancer cell treatment, whereas F4.6.3 exhibits advanced wound healing properties.

## 4. Conclusions

A spindle-damaging activity became enriched by fractionations, which was reflected by increased *α*-tubulin acetylation followed by the activation of caspases 8 and 3 and the typical nuclear morphology of apoptosis. The impact on apoptosis and the orchestration of apoptosis regulator activation were similar for both fractions. In comparison to earlier work, genotoxic components activating Chk2 were eliminated.

Importantly, F4.6.3 induced mobility markers, whereas F4.3.7 inhibited the expression of mobility markers or did not interfere with their constitutive expression. This property implicates an inhibitory effect of F4.3.7 on tumour progression which was also reflected by the downregulation of the Jun family of oncogenes and the suppression of Stat3 activity.

These findings can provide a basis for the development of remedies (1) supporting wound healing in case of F4.6.3 or (2) interfering with tumour progression in case of F4.3.7.

## Figures and Tables

**Table 1:** Mobile phases used for VLC of the dichloromethane extract.

Mobile phase	Relation	Volume (l)	Fractions	Residue (g)
Petroleum ether		4		
Petroleum ether : chloroform	9 : 1	5	**F1.1 **	23.9
Chloroform		12		
Chloroform : methanol	9 : 1	9	F1.2	10.8 g
Chloroform : methanol	7 : 3	9		
Chloroform : methanol	5 : 5	9		
Chloroform : methanol	3 : 7	9	F1.3	1.6 g
Chloroform : methanol	1 : 9	9		
Methanol		9		

**Table 2:** Mobile phases used for VLC of F1.1.

Mobile phase	Relation	Volume (l)	Fractions	Residue (g)
Dichloromethane : hexan	8 : 2	2	F2.1	1.63
Dichloromethane		2	F2.2	0.17
Dichloromethane : ethyl acetate	8 : 2	1	F2.3	1.28
Dichloromethane : ethyl acetate	6 : 4	1	F2.4	
Dichloromethane : ethyl acetate	4 : 6	1	F2.5	4.57
Dichloromethane : ethyl acetate	2 : 8	1	**F2.6**	1.73
Ethyl acetate		1	F2.7	0.22
Ethyl acetate : methanol	8 : 2	1	F2.8	0.04
Ethyl acetate : methanol	6 : 4	1	F2.9	0.12

**Table 3:** Mobile phases used for CC of F2.6.

Mobile phase	Relation	Volume (l)	Fractions	Residue (g)
Dichloromethane	100%	1	F3.1	0.02
Dichloromethane : ethyl acetate	80 : 20	2	F3.2	0.05
			**F3.3**	1.14
Dichloromethane : ethyl acetate	60 : 40	1	F3.4	0.07
			F3.5	0.05
Dichloromethane : ethyl acetate	40 : 60	1	**F3.6 **	0.32
			F3.7	0.02
Dichloromethane : ethyl acetate	20 : 80	1	F3.8	0.01
			F3.9	0.03
Ethyl acetate	100%	1	F3.10	0.04

**Table 4:** Mobile phases used for CC of F3.3.

Mobile phase	Relation	Volume (mL)	Fractions	Residue (mg)
Dichloromethane	100%	500	F4.3.1	0.4
Dichloromethane : ethyl acetate	90 : 10	500	F4.3.2	3
			F4.3.3	59
			F4.3.4	91
Dichloromethane : ethyl acetate	80 : 20	500	F4.3.5	256
			F4.3.6	65
Dichloromethane : ethyl acetate	60 : 40	500	**F4.3.7 **	146
			F4.3.8	8
Dichloromethane : ethyl acetate	40 : 60	500	F4.3.9	6
			F4.3.10	111
Dichloromethane : ethyl acetate	20 : 80	500	F4.3.11	63
Ethyl acetate	100%	500	F4.3.12	3

**Table 5:** Stationary phase, mobile phases, and detection methods used for TLC.

Stationary phase	Silica gel plates 60 F254 (Merck, Darmstadt, Germany)
Mobile phase	TLC system 1 : chloroform : methanol : water 90 : 22 : 3.5
	TLC system 2 : chloroform : methanol : water 90 : 3.5 : 0.2
	TLC system 3 : dichloromethane : ethyl acetate 80 : 20
	TLC system 4 : dichloromethane : ethyl acetate 85 : 15
	TLC system 5 : chloroform : methanol : water 70 : 22 : 3.5
Detection	UV_254, _UV_365_, visible light
	Anisaldehyde sulphuric acid reagent (ASR)
